# Association of modifiable lifestyle with colorectal cancer incidence and mortality according to metabolic status: prospective cohort study

**DOI:** 10.3389/fonc.2023.1162221

**Published:** 2023-05-30

**Authors:** Peng Xie, Siqing Wu, Zichong Kuo, Huidong Tian, Qiangsheng He, Yanfei Li, Ningning Mi, Linmin Hu, Haitong Zhao, Wenjing Li, Bin Xia, Jinqiu Yuan, Kehu Yang, Changhua Zhang, Yulong He

**Affiliations:** ^1^Digestive Diseases Center, The Seventh Affiliated Hospital, Sun Yat-sen University, Shenzhen, China; ^2^Guangdong Provincial Key Laboratory of Digestive Cancer Research, The Seventh Affiliated Hospital of Sun Yat-sen University, Shenzhen, Guangdong, China; ^3^School of Medicine, Sun Yat-sen University, Shenzhen, China; ^4^School of Public Health, Guangzhou Medical University, Guangzhou, China; ^5^Clinical Research Center, Big Data Center, The Seventh Affiliated Hospital, Sun Yat-sen University, Shenzhen, Guangdong, China; ^6^Evidence Based Social Science Research Center, School of Public Health, Lanzhou University, Lanzhou, China; ^7^The First School of Clinical Medicine, Lanzhou University, Lanzhou, Gansu, China; ^8^Department of General Surgery, The First Hospital of Lanzhou University, Lanzhou, Gansu, China; ^9^School of Public Health, Sun Yat-sen University, Shenzhen, China

**Keywords:** CRC, healthy lifestyle, metabolic syndrome, incidence, mortality

## Abstract

**Background:**

Metabolic syndrome has been linked to an increased risk of colorectal cancer (CRC) incidence and mortality, but whether adopting a healthy lifestyle could attenuate the risk of CRC conferred by metabolic syndrome remains unclear. The aim of the study is to investigate the individual and joint effects of modifiable healthy lifestyle and metabolic health status on CRC incidence and mortality in the UK population.

**Methods:**

This prospective study included 328,236 individuals from the UK Biobank. An overall metabolic health status was assessed at baseline and categorized based on the presence or absence of metabolic syndrome. We estimated the association of the healthy lifestyle score (derived from 4 modifiable behaviors: smoking, alcohol consumption, diet, physical activity and categorized into “favorable,” “intermediate”, and “unfavorable”) with CRC incidence and mortality, stratified by metabolic health status.

**Results:**

During a median follow-up of 12.5 years, 3,852 CRC incidences and 1,076 deaths from CRC were newly identified. The risk of incident CRC and its mortality increased with the number of abnormal metabolic factors and decreased with healthy lifestyle score (P trend = 0.000). MetS was associated with greater CRC incidence (HR = 1.24, 95% CI: 1.16 – 1.33) and mortality (HR = 1.24, 95% CI: 1.08 – 1.41) when compared with those without MetS. An unfavorable lifestyle was associated with an increased risk (HR = 1.25, 95% CI: 1.15 – 1.36) and mortality (HR = 1.36, 95% CI: 1.16 – 1.59) of CRC across all metabolic health status. Participants adopting an unfavorable lifestyle with MetS had a higher risk (HR = 1.56, 95% CI: 1.38 – 1.76) and mortality (HR = 1.75, 95% CI: 1.40 – 2.20) than those adopting a favorable healthy lifestyle without MetS.

**Conclusion:**

This study indicated that adherence to a healthy lifestyle could substantially reduce the burden of CRC regardless of the metabolic status. Behavioral lifestyle changes should be encouraged for CRC prevention even in participants with MetS.

## Introduction

CRC is one of the most diagnosed malignancies worldwide, leading to nearly 1 million deaths per year (1). Both hereditary and environmental risk factors contribute to the development of CRC. Although the etiology of CRC has not been fully elucidated, accumulating epidemiologic evidence have suggested that metabolic syndrome (MetS) is the major risk factor for CRC incidence and mortality (2, 3). MetS and its related factors, including central obesity, hypertension, hyperglycemia, low HDL cholesterol and hyperlipidemia that might independently contribute to processes like angiogenesis and oxidative stress, could potentiate the risk of CRC (4–8). Therefore, a global trend with highly prevalent metabolic syndrome components may lead to a heavier CRC burden (9–11).

Since primary prevention based on risk factors of CRC may have the lowest cost and best effect among all strategies, then lifestyle modification is essential. Indeed, unhealthy lifestyle, including smoking (12, 13), alcoholic consumption (14), poor diet (15, 16), and physical inactivity (17) have been linked with the increased risk and mortality of CRC. Individuals with MetS may suffer a higher risk of CRC incidence and mortality (2, 18, 19), whether the increased risk could be offset by a healthy lifestyle remains hypothetical. No studies have systematically assessed the relationship of combined lifestyle factors with the risk of CRC incidence and mortality across a population at different degrees of metabolic risk.

We performed this prospective analysis based on the UK biobank database to assess the individual and joint effect of metabolic risk and healthy lifestyle on the risk of CRC incidence and mortality. We further examined the beneficial reduction in CRC risk provided by these modifiable factors across groups stratified by metabolic risk for CRC.

## Materials and methods

### Study design and study population

UK biobank is a large, prospective cohort study based on over 0.5 million participants recruited from 2006 to 2010 across England, Wales, and Scotland. Details about study design and information extraction have been reported previously (20). The UKB cohort was approved by North West Multicenter Research Ethics Committee. Written informed consent was obtained from all participants before the study.

In the study, participants with any diagnosed malignancy prior to baseline (except for nonmelanoma skin cancer, n = 26,819), or with missing data on lifestyle or other covariates (n = 146,172) were excluded, leaving 328,236 participants in the final analysis (**Figure S1).**

### Assessment of outcome

CRC cases were identified through linkage to cancer and death registries by using the International Classification of Diseases, 10th Revision (ICD-10). The endpoints of present study were incidence of and mortality from CRC (ICD-10 C18-C20). Eligible patients contributed person-years from recruitment date until date of CRC diagnosis, date of death, date of withdrawal from the study, or last date of follow-up (31 Dec 2021), whichever came first.

### Assessment of healthy lifestyle factors

In order to assess adherence to a healthy lifestyle, based on previous knowledge (21–23), 4 factors (smoking, alcohol consumption, diet, physical activity) were used to construct healthy lifestyle score, excluding body mass index (BMI), as it is strongly related to MetS. Based on the World Cancer Research Fund/American Institute of Cancer Research [WCRF/AICR] recommendations, smoking was defined as ideal if individuals were non-smokers, poor intermediate if previous smokers, or poor if current smokers. As for alcohol, the ethanol content was calculated, and then converted into standard units (g/d) (24). Thereafter, alcohol was considered as ideal if individuals were non-drinker (0 g/d), intermediate if moderate alcohol consumers (0 < n ≤ 28 g/d for males and n ≤ 14 g/d for females, or poor if excess alcoholic drinker (n > 28 g/d for males and n > 14 g/d for females) (25). Physical activity was assessed using the International Physical Activity Questionnaire (IPAQ) (26, 27), and then grouped into three groups: low (0 < n ≤ 600 MET minutes/week), moderate (600 < n ≤ 3000 MET minutes/week), high (n ≥ 3000 MET minutes/week) (28, 29). A food frequency questionnaire was used to obtain dietary information. According to previous studies (15, 30, 31), 4 main food components (whole grains, vegetables, fruits, red and processed meats) that have associations with CRC are used to compose a diet score which is then categorized into 3 groups (favorable, intermediate, unfavorable).

A healthy lifestyle score (HLS) was created based on recommendations that may confer some benefit (25). For smoking, alcohol consumption and physical activity, 1 point was assigned for high, 0.5 point for mediate, 0 point for low. Total score for diet was 2 points, as it included several factors. Details about point assignment could be seen in **SUPPLEMENT 1**. A healthy lifestyle score was subsequently categorized into 3 groups: favorable (n ≥ 3.5 points), intermediate (3.5 points > n ≥ 2.75 points) and unfavorable (n < 2.75 points).

### Assessment of metabolic status

The metabolic status was categorized two groups according to the presence or absence of metabolic syndrome. Metabolic syndrome (MetS) includes at least 3 component as mentioned below (4): (1) waist circumference (WC) of ≥ 85 cm in women or ≥ 90 cm in men; (2) fasting plasma glucose of ≥ 100 mg/dL or ongoing drug treatment for diabetes mellitus (DM); (3) blood pressure of ≥130/85 mmHg or ongoing drug treatment for hypertension; (4) serum HDL-C of < 50 mg/dL in women or < 40 mg/dL in men; and (5) serum triglyceride of ≥ 150 mg/dL.

### Assessment of covariates

Information on sociodemographic characteristics, health and medical history and lifestyle factors was collected through touchscreen questionnaire at baseline, including age, ethnicity, gender, index of multiple deprivation (IMD), family history of cancer, sleep time, medication use [non-aspirin non-steroidal anti-inflammatory drugs (NSAIDs), vitamin supplement, mineral supplement, aspirin]. Anthropometric data, including height, body weight, and waist circumference was measured in the assessment center. The components of abnormal metabolism were measured during physical examination. Further details on the derivation of these variables could be seen via the website (http://www.ukbiobank.ac.uk).

### Statistical analysis

Cox proportional hazards models were performed to evaluate the effect of metabolic status, healthy lifestyle and their combination on the risk of CRC incidence and mortality. Hazard ratios (HRs) with 95% confidence intervals (CIs) were calculated for lifestyle categories within each metabolic status stratum. Cox models were adjusted for age, gender, assessment center, ethnicity, family history of cancer, sleep time, use of vitamin supplement, use of mineral supplement, aspirin, use of non-aspirin NSAIDs, and use of statin. The interaction between metabolic status and healthy lifestyle score was tested by adding an interaction term in the Cox regression models.

To investigate possible effect modification, we conducted additional stratified analyses according to gender, age, history of bowel screening and family history of cancer. In addition, we performed several sensitivity analyses to validate the robustness of the main findings. Firstly, we excluded participants who developed CRC or died during the first two years of follow-up to minimize reverse causality. Secondly, we used competing risk regression to account for the competing risk of death, and diagnosis of any other cancer (except for non-melanoma skin cancer). All statistical analyses were performed using R software (version 3.5.3, R Foundation for Statistical Computing, Vienna, Austria) and a two-sided *P*-value of < 0.05 was defined as a significant difference.

## Results

### Baseline characteristics of study population

In total, 328,236 participants were finally included in the final analysis. **Table 1** shows better the baseline characteristics of the participants by the healthy lifestyle categories. The study population had a mean age of 56 years with 48% males. Compared with individuals adopting an unfavorable lifestyle (24%, n = 79,405), individuals with an intermediate or favorable healthy lifestyle (76%, n = 248,831) tended to be female, with a lower WC, a better general health condition, a higher prevalence of MetS, long standing illness, and medication use (aspirin, NASIDs, and statin). During a median follow-up of 12.5 years, 3,852 incidences of CRC and 1,076 deaths were registered.

**Table 1:** Demographics, lifestyle and metabolic characteristics of included participants.

	Unfavorable (n=79405)	Intermediate (n=129975)	Favorable (n=118856)
White Ethnicity (%)	76070 (96.1)	123998 (95.6)	110337 (93.1)
Mean (SD) Age (years)	55.40 (8.11)	55.90 (8.14)	56.54 (8.08)
Male (%)	47323 (59.6)	63159 (48.6)	47981 (40.4)
Mean (SD) BMI (kg/m^2^)	27.82 (4.83)	27.48 (4.73)	26.84 (4.58)
Mean (SD) WC (cm)	93.44 (13.51)	90.66 (13.34)	87.73 (12.98)
Mean (SD) IMD^*^ (%)	18.47 (14.93)	16.15 (13.23)	16.47 (13.27)
Excellent Overall Health Rating (%)	9356 (11.8)	22831 (17.6)	26610 (22.4)
Long Standing Illness (%)	27122 (34.2)	38559 (29.7)	33928 (28.5)
Use of Aspirin (%)	12902 (16.2)	17994 (13.8)	15320 (12.9)
Use of NASIDs^#^ (%)	14002 (17.6)	21059 (16.2)	18214 (15.3)
Use of Statin (%)	14300 (18.0)	20302 (15.6)	17157 (14.4)
Use of Vitamin (%)	10399 (13.1)	18567 (14.3)	20157 (17.0)
Use of Mineral (%)	14641 (18.5)	27121 (20.9)	29504 (24.9)
Family History of Cancer			
Yes	28057 (35.3)	45776 (35.2)	41426 (34.9)
No	51348 (64.7)	84199 (64.8)	77430 (65.1)
Smoking Status (Points) (%)			
0	31036 (39.1)	6416 (4.9)	469 (0.4)
0.5	11219 (14.1)	9453 (7.3)	2682 (2.3)
1	37150 (46.8)	114106 (87.8)	115705 (97.3)
Alcoholic Drinking (Points) (%)			
0	35638 (44.9)	27098 (20.8)	6287 (5.3)
0.5	42244 (53.2)	97372 (74.9)	94327 (79.4)
1	1523 (1.9)	5505 (4.2)	18242 (15.3)
Physical Training (Points) (%)			
0	36240 (45.6)	23279 (17.9)	2459 (2.1)
0.5	34637 (43.6)	82087 (63.2)	48822 (41.1)
1	8528 (10.7)	24609 (18.9)	67575 (56.9)
Diet (Points) (%)			
0.25	6342 (8.0)	1015 (0.8)	87 (0.1)
0.5	21042 (26.5)	20175 (15.5)	433 (0.4)
0.75	33929 (42.7)	28097 (21.6)	14472 (12.2)
1	10576 (13.3)	54890 (42.2)	19646 (16.5)
1.25	6671 (8.4)	15188 (11.7)	45764 (38.5)
1.5	706 (0.9)	10169 (7.8)	29592 (24.9)
1.75	139 (0.2)	422 (0.3)	8518 (7.2)
2	0 (0.0)	19 (0.0)	344 (0.3)
Metabolic Syndrome Components (%)			
0	9070 (11.4)	18135 (14.0)	18884 (15.9)
1	19935 (25.1)	36604 (28.2)	36773 (30.9)
2	22110 (27.8)	34902 (26.9)	30855 (26.0)
3	16630 (20.9)	24163 (18.6)	19624 (16.5)
4	8863 (11.2)	12328 (9.5)	9810 (8.3)
5	2797 (3.5)	3843 (3.0)	2910 (2.4)

^*^IMD, index of multiple deprivation.

^#^NSAIDs, non-aspirin non-steroidal anti-inflammatory drugs.

### Association of metabolic status with CRC incidence and mortality

**Figure 1:**
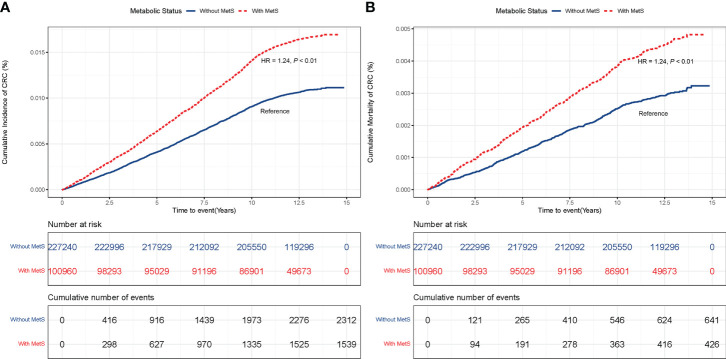
**(A)** CRC incidence in participants with and without MetS. **(B)** CRC mortality in participants with and without MetS.

The risk of CRC incidence and mortality were monotonically associated with the number of abnormal metabolic components (*P*_trend_ < 0.01; **Figures S2A, B**). Compared with those without MetS, participants with MetS had a higher risk of CRC incidence (HR = 1.24, 95% CI: 1.16 – 1.33, *P* < 0.01) (**Figure 1A**), and mortality (HR = 1.24, 95% CI: 1.08 – 1.41, *P* < 0.01) (**Figure 1B**). These results were unchanged after adjusting for lifestyle factors (**Table S1A**), revealing that metabolic status was independently associated with the risk of CRC incidence and mortality.

### Association of healthy lifestyle and CRC incidence and mortality

**Figure 2:**
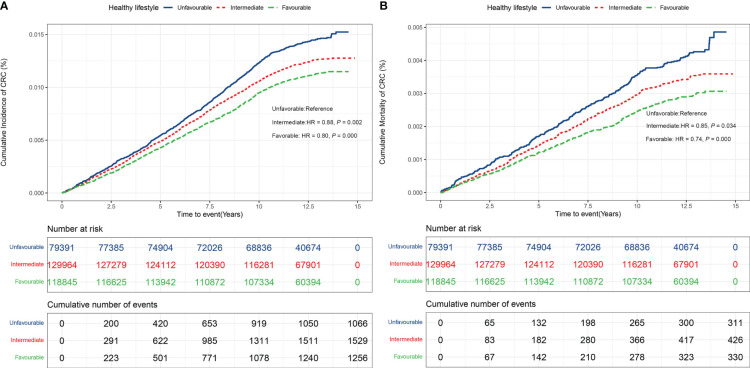
**(A)** CRC incidence in participants stratified by healthy lifestyle (Unfavorable, Intermediate, Favorable). **(B)** CRC mortality in participants stratified by healthy lifestyle (Unfavorable, Intermediate, Favorable).

As shown in **Figures 2A, B** adopting a healthy lifestyle was associated with a lower risk of CRC incidence and mortality. The relative risk of CRC incidence and mortality were lower in participants with an intermediate (incidence: HR = 0.88, 95% CI: 0.81 – 0.95; mortality: HR = 0.85, 95% CI: 0.73 – 0.99, respectively), and favorable lifestyle (HR 0.80, 95% CI: 0.74 – 0.87; HR = 0.74, 95% CI: 0.63 – 0.86, respectively), as compared with participants with unfavorable lifestyle. These estimates were unchanged after adjusting for metabolic status, indicating that lifestyle was associated with CRC risk independently (**Table S1B**). The same pattern of results was noted when the number of healthy lifestyle factors was used instead of lifestyle categories (**Figures S3A, B**).

### Joint effect of healthy lifestyle and metabolic status on the risk and mortality of CRC

**Table 2** shows the associations of adopting a healthy lifestyle with the risk of CRC incidence and mortality stratified by metabolic status. Adopting an intermediate or favorable lifestyle was associated with the lower risk of incident CRC across subgroups of population defined by baseline metabolic status (**Table 2**).

**Table 2:** Associations between Healthy lifestyle, Metabolic Status and CRC.

	Cases/Person-years	Hazard ratio (95% CI)	*P-interaction*
**CRC incidence**			0.45
With MetS			
Unfavorable lifestyle	482/326346	1.00[Reference]	
Intermediate lifestyle	597/472123	0.87[0.77, 0.99]	
Favorable lifestyle	461/380768	0.86[0.75, 0.98]	
Without MetS			
Unfavorable lifestyle	584/605996	1.00[Reference]	
Intermediate lifestyle	932/1079042	0.89[0.81, 0.99]	
Favorable lifestyle	796/1043185	0.79[0.71, 0.88]	
**CRC mortality**			0.43
With MetS			
Unfavorable lifestyle	144/344043	1.00[Reference]	
Intermediate lifestyle	161/497524	0.78[0.62, 0.98]	
Favorable lifestyle	121/400040	0.75[058, 0.96]	
Without MetS			
Unfavorable lifestyle	167/632742	1.00[Reference]	
Intermediate lifestyle	265/1123728	0.92[0.75, 1.12]	
Favorable lifestyle	209/1084260	0.75[0.61, 0.92]	

Similarly, we found that participants adopting a favorable lifestyle had a 25% lower risk of CRC and mortality as compared to participants with an unfavorable lifestyle across all metabolic status subgroups (HR = 0.75, 95% CI: 0.58 – 0.96; HR = 0.75, 95% CI: 0.61 – 0.92, respectively). Compared with an unfavorable lifestyle, intermediate lifestyle was associated with a 22% reduction in risk of CRC mortality in participants with MetS (HR = 0.78, 95% CI: 0.62 – 0.98), but was not associated with CRC mortality in participants without MetS (HR = 0.92, 95% CI: 0.75 – 1.12).

**Figure 3:**
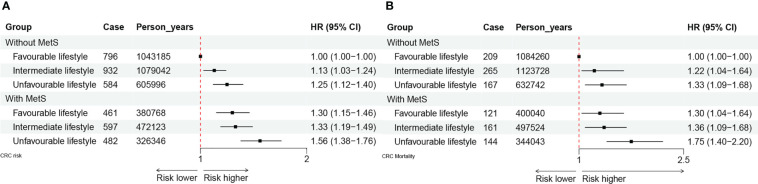
**(A)** The joint effect of healthy lifestyle and metabolic status on CRC incidence. **(B)** The joint effect of healthy lifestyle and metabolic status on CRC mortality.

We also noted a joint effect of metabolic and lifestyle factors on the risk of CRC incidence (**Figure 3A**) and mortality (**Figure 3B**). Participants with MetS and an unfavorable lifestyle had the highest risk of CRC incidence and mortality as compared with those without MetS and a favorable lifestyle (HR = 1.56, 95% CI: 1.38 – 1.76; HR = 1.75, 95% CI: 1.40 – 2.20, respectively).

These observed pattern of associations of metabolic status and healthy lifestyle factors with the risk of CRC incidence and mortality did not differ by gender (P = 0.11; P = 0.26), history of bowel screening (P = 0.11; P = 0.37), age (P = 0.73; P = 0.10), and family history of cancer (P = 0.88; P = 0.55) (**Table S2**). These estimated effect size remain essentially unchanged in several sensitivity analyses that excluded the events occurred during the first 2 years of follow-up, and using the competing risk proportional subdistribution hazards modes (**Table S3**).

## Discussion

In this prospective cohort study, we investigated the individual and joint effect of metabolic risk and healthy lifestyle on the risk of CRC incidence and mortality using over 340,000 participants in the UK Biobank. MetS could increase 24% overall risk of CRC incidence and mortality compared with those without MetS regardless of healthy lifestyle. Adopting an unfavorable lifestyle could further increase CRC incidence and mortality by 20% and 26% respectively when compared with a favorable lifestyle regardless of metabolic status. Participants with MetS and an unfavorable lifestyle profile had a nearly 1.5- and 2- fold risk of CRC incidence and mortality compared to those without MetS and a favorable lifestyle.

Consistent with our findings, previous studies have demonstrated that MetS is associated with a higher risk of CRC incidence and mortality (32–35). For example, a recent nested case–control study in the U.S. showed that MetS was associated with a 25% higher risk of early-onset CRC (HR = 1.25; 95% CI 1.09 – 1.43) (33). A meta-analysis involving 18 studies with CRC incidence and 12 studies with CRC mortality found that patients with MetS had a 25% higher CRC incidence, and a 15% higher CRC mortality (34). However, many established risk factors for CRC, like family history of CRC, history of colonoscopy, smoking, alcohol consumption and diet were not always fully controlled for in most previous studies (33–35), which might lead to residual confounding. In present study, we adjusted for known risk factors for CRC and found that MetS was independently associated with the risk of CRC incidence and mortality. Consistent findings from diverse populations and different studies implied the importance of MetS on the aetiology and preventative strategies of CRC.

We also found that adopting a favorable lifestyle could decrease the risk of CRC incidence and mortality among patients with or without MetS after adjusting for known risk factors, which were in agreement with previous epidemiological studies (36–39). For example, a large cohort study based on the Nurses’ Health Study and the Health Professionals Follow-up Study suggested that adherence to a healthy lifestyle was associated with a reduced CRC incidence and mortality regardless of endoscopic screening (36). Another study based on the DACHS (Darmkrebs Chancen der Verhu¨tung durch Screening) study found that a healthy lifestyle score was increasingly associated with a lower risk of CRC independent of patient’s genetic predisposition (37). However, although importance of screening in preventing CRC has been recognized (40), whether adhering to a healthy lifestyle could attenuate the increased risk caused by MetS remains unclear. To our knowledge, this is the first to examine the joint beneficial association of lifestyle and metabolic health factors with CRC incidence and mortality. The present study has bridged this gap so it would be nice to really emphasize this important new knowledge.

Several previous studies suggested that the associations of lifestyle factors with the risk of CRC incidence and mortality may vary by an individual’s metabolic risk status (41). A prospective cohort study in the United States indicated that moderate to vigorous metabolic status physical activity was associated with a reduced risk of colon cancer in non-diabetics, but not in diabetic patients (41). The present study did not find sufficient evidence that the association of lifestyle factors with CRC risk could be modified by metabolic status. We did observe a synergistic effect of lifestyle and metabolic factors with CRC incidence and mortality, which was in line with previous findings (42). For example, a case-control study in China found that patients with an unfavorable lifestyle and a high level of comorbidity risk had a 10.33-fold increased CRC risk (OR = 10.33, 95% CI: 6.59 – 16.18) (42). These findings, including ours, suggested that people belonging to this group had a higher risk of CRC and require targeted support and services.

Mechanisms linking MetS and CRC incidence had been partly elucidated before. Insulin resistance induced by MetS might promote carcinogenesis through insulin, insulin-like growth factor 1 signaling, and systemic inflammation (6, 43, 44). Recent researches also suggested that the gut microbiota might promote cancer development by modulating the bile acid-microbiota crosstalk and microbe-derived proinflammatory molecules, like lipopolysaccharide (45, 46). Factors included in the healthy lifestyle, like physical training and diet, is helpful to prevent central obesity, and to restore microbiota dysfunction in rats (47, 48). Based on current knowledge we have before concluding this study, we hypothesized that adopting a healthy lifestyle could decrease the risk and mortality by partly intervening or reversing MetS. However, the reduction in HR of risk and mortality of CRC was almost the same in both population with or without MetS, and no multiplicative interactions between healthy lifestyle and metabolic status were identified, which indicate that a healthy lifestyle might act through additional mechanisms. Further investigations would be necessary to clarify these connections.

The major strengths of the present study include the study of a healthy lifestyle and metabolic status in the large sample size of the UK Biobank. Furthermore, we studied the combined effects of metabolic status and healthy lifestyle, systematically investigated the association between them and risk, mortality of CRC, and provided solid evidence for CRC prevention and prognosis in individuals with varied metabolic status. The present study has several limitations. Firstly, some data were not available, the influence of other pathogenic factors on CRC like genetic predisposition could not be calculated in the study, and the risk of CRC may not be so specific. Secondly, due to the limitations of the UK biobank, the findings may not be generalizable to other ethnic groups, and as such, large-scale studies involving other races and ethnicities may be required to confirm the conclusions. Thirdly, lifestyle factors were self-reported, hence the lifestyle risk levels could be misclassified.

## Conclusion

In summary, associations of a healthy lifestyle with the risk of CRC in individuals with varied metabolic health have been analyzed quantitively in a large cohort study. The findings show that adopting a healthy lifestyle could decrease the overall risk and mortality of CRC, even in individuals with MetS.

## Data availability statement

Publicly available datasets were analyzed in this study. This data can be found here: https://www.ukbiobank.ac.uk/.

## Author contributions

BX contributed to conception and design of the study. JY organized the database and performed the statistical analysis. PX wrote the first draft and QH, BX modified the manuscript. ZK checked for possible linguistic problems. All authors contributed to the article and approved the submitted version.
