# Supplementary figures and images for: Association of modifiable lifestyle with colorectal cancer incidence and mortality according to metabolic status: prospective cohort study
Source: Front Oncol. 2023 May 30;13:1162221. doi: 10.3389/fonc.2023.1162221 (PMC10262687; doi:10.3389/fonc.2023.1162221)

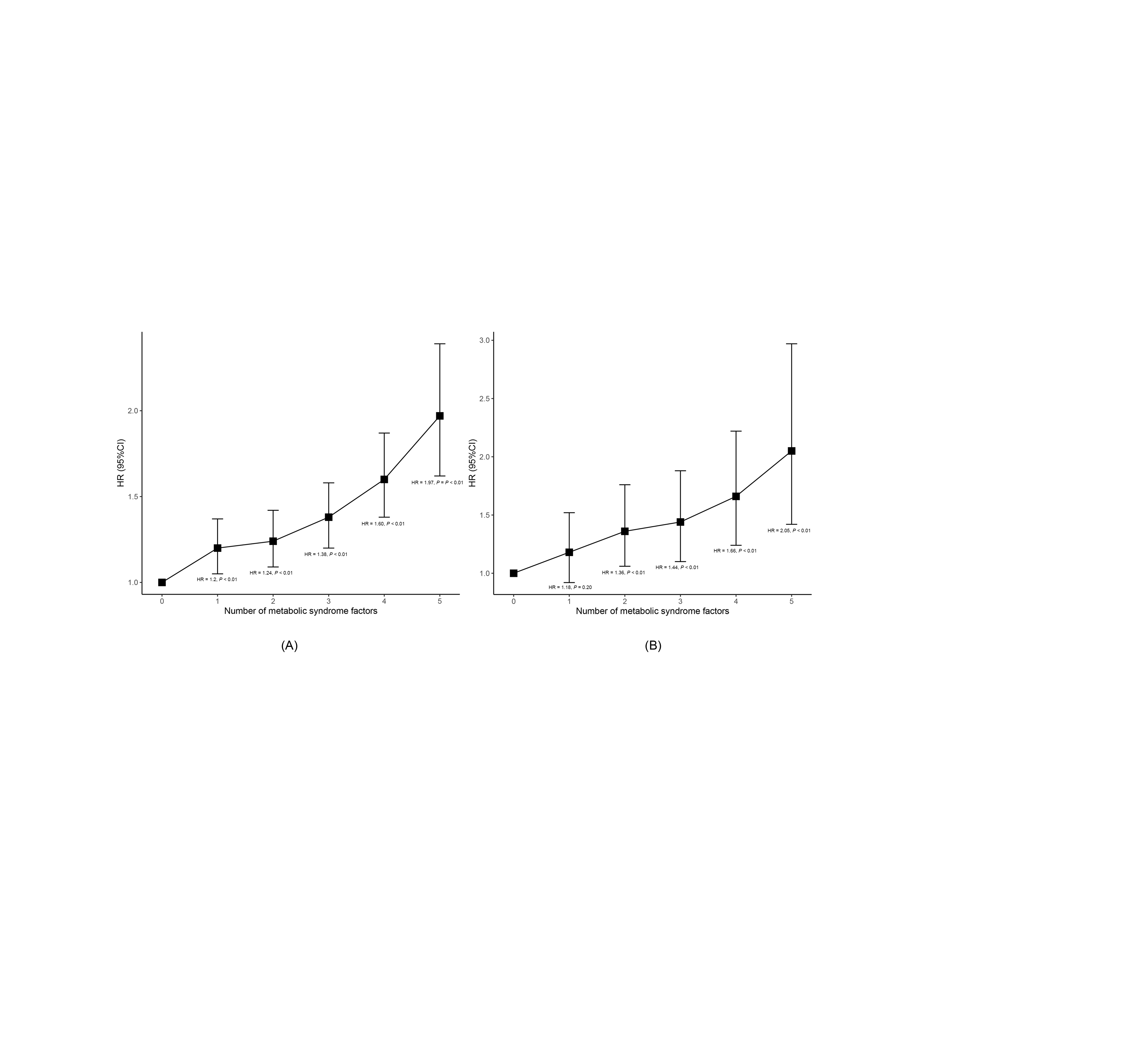

Supplement: Supplementary file 1 [file Image_1.tif]

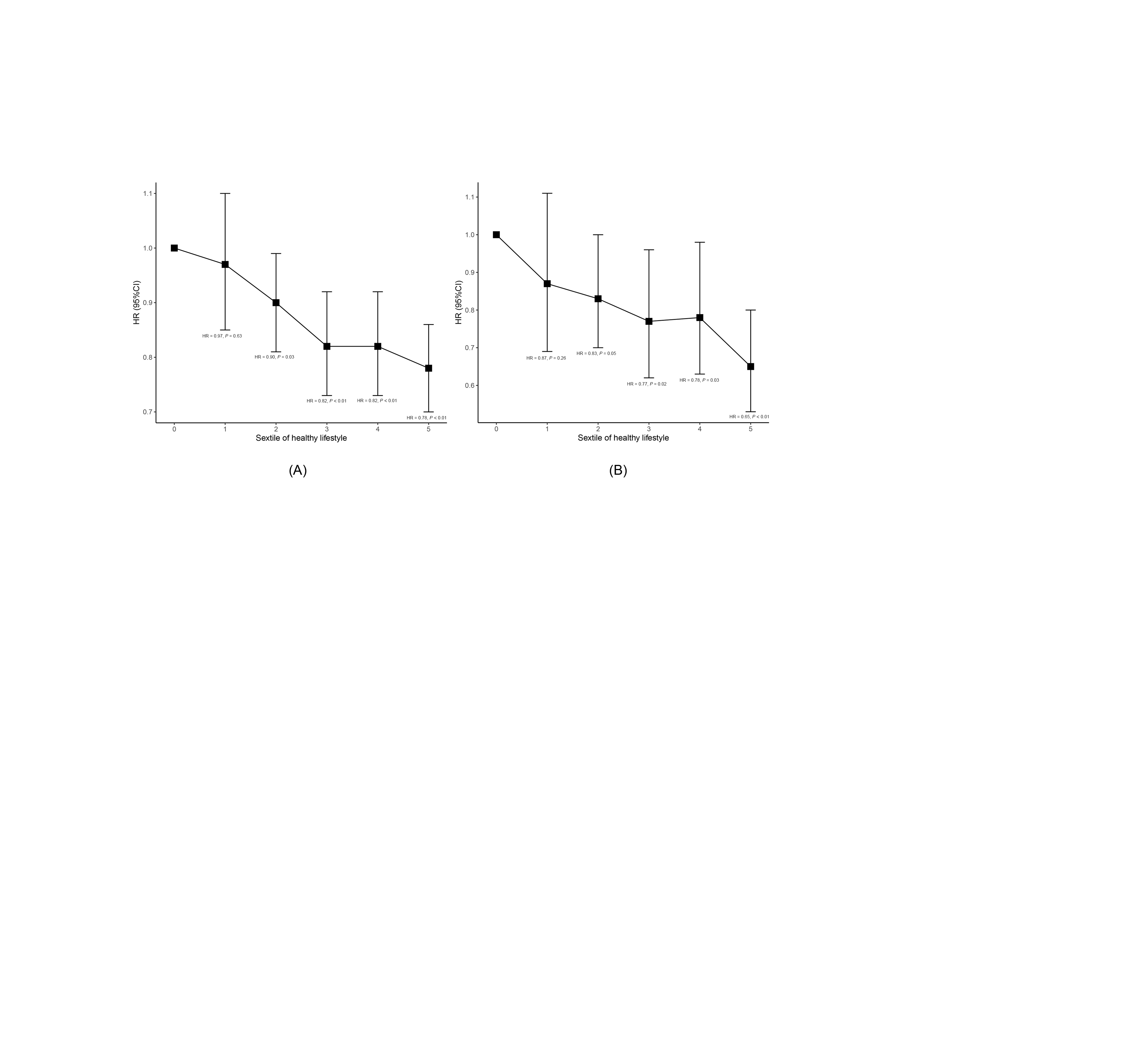

Supplement: Supplementary file 2 [file Image_2.tif]

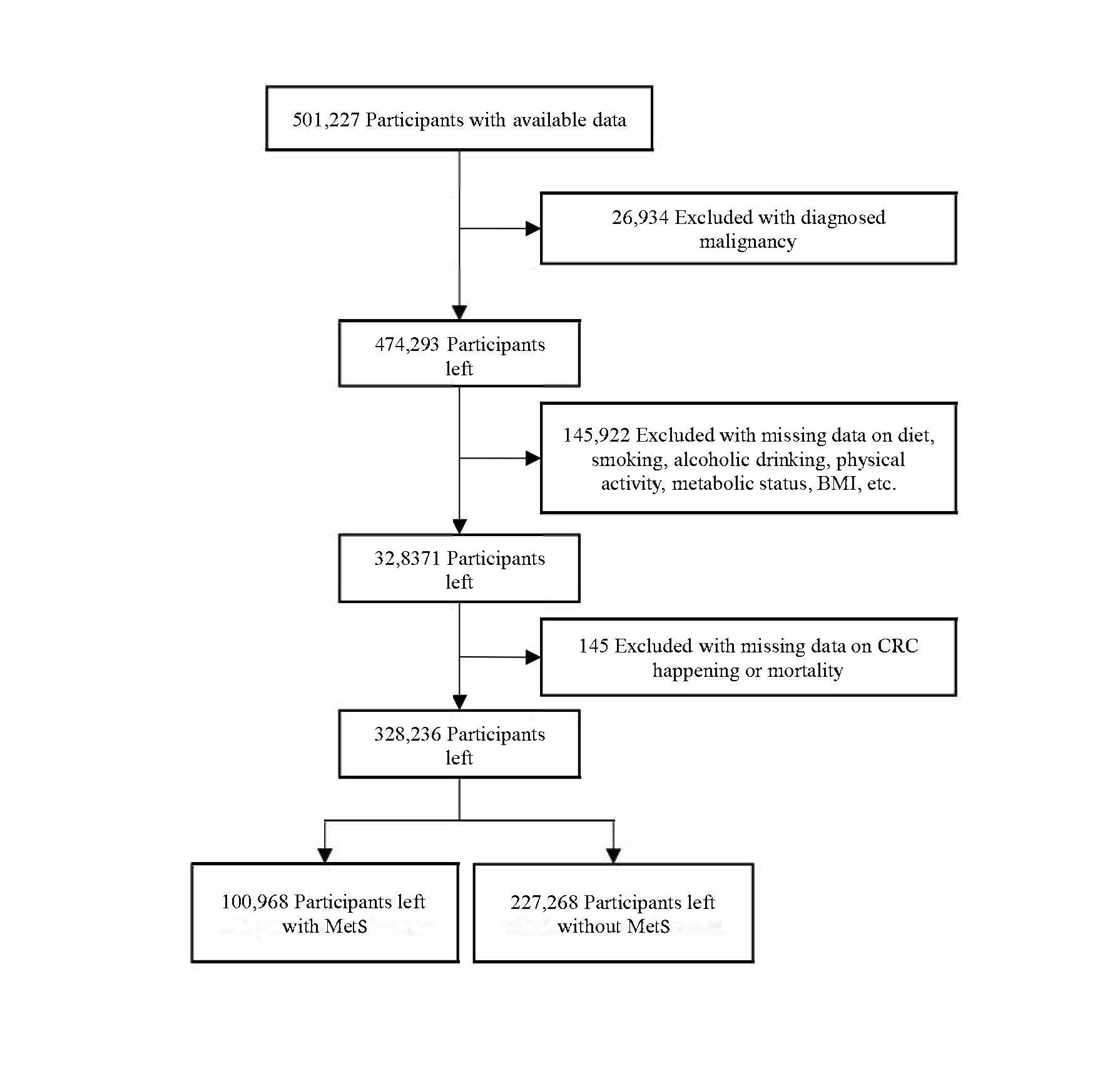

Supplement: Supplementary file 3 [file Image_3.tif]
