# Supplementary material for: Association of modifiable lifestyle with colorectal cancer incidence and mortality according to metabolic status: prospective cohort study
Source: Front Oncol. 2023 May 30;13:1162221. doi: 10.3389/fonc.2023.1162221 (PMC10262687; doi:10.3389/fonc.2023.1162221)
Supplement: Supplementary file 4 [file Table_1.docx]

| Table S1A. Associations between metabolic status and CRC | | | |
| --- | --- | --- | --- |
|  |  | Hazard ratio (95% CI) | |
|  | Cases/ Person-years | Model 1† | Model 2‡ |
| **CRC incidence** |  |  |  |
| Metabolic ideal | 2312/2728223 | 1.00[Reference] | 1.00[Reference] |
| Metabolic poor | 1539/1179237 | 1.24[1.16, 1.33] | 1.23[1.15, 1.32] |
| **CRC mortality** |  |  |  |
| Metabolic ideal | 641/2840730 | 1.00[Reference] | 1.00[Reference] |
| Metabolic poor | 426/1241607 | 1.24[1.08, 1.41] | 1.23[1.07, 1.40] |

Abbreviation: HR, hazard ratio.
† Model 1: Cox proportional hazards regression adjusted for age, sex, socioeconomic status (index of multiple deprivation, fifth), family history of cancer and other covariates;
‡ Model 2: Additionally, mutually adjusted for metabolic status based on model 1.

| Table S1B. Associations between healthy lifestyle and CRC | | | |
| --- | --- | --- | --- |
|  |  | Hazard ratio (95% CI) | |
|  | Cases/ Person-years | Model 1† | Model 2‡ |
| **CRC incidence** |  |  |  |
| Favorable lifestyle | 1256/1423953 | 1.00[Reference] | 1.00[Reference] |
| Intermediate lifestyle | 1529/1551165 | 1.10[1.02, 1.18] | 1.09[1.01, 1.18] |
| Unfavorable lifestyle | 1066/932342 | 1.25[1.15, 1.36] | 1.23[1.13, 1.34] |
| **CRC mortality** |  |  |  |
| Favorable lifestyle | 330/1484300 | 1.00[Reference] | 1.00[Reference] |
| Intermediate lifestyle | 426/1621252 | 1.16[1.00, 1.34] | 1.15[0.99, 1.33] |
| Unfavorable lifestyle | 311/976786 | 1.36[1.16, 1.59] | 1.35[1.15, 1.58] |

Abbreviation: HR, hazard ratio.
† Model 1: Cox proportional hazards regression adjusted for age, sex, socioeconomic status (index of multiple deprivation, fifth), family history of cancer and other covariates;
‡ Model 2: Additionally, mutually adjusted for metabolic status based on model 1.

Table S2 Subgroup analysis stratified by age, gender, history of bowel screening, and family history of cancer

| Subgroup analysis | CRC incidence | | | CRC mortality | | |
| --- | --- | --- | --- | --- | --- | --- |
|  | Hazard ratio (95% CI) | | *P-interaction* | Hazard ratio (95% CI) | | *P-interaction* |
| **Age** | ＞55 years | ≤55 years | 0.73 | ＞55 years | ≤55 years | 0.10 |
| Metabolic ideal + Favorable lifestyle | 1.00 [Reference] | 1.00 [Reference] |  | 1.00 [Reference] | 1.00 [Reference] |  |
| Metabolic ideal + Intermediate lifestyle | 1.14 [1.02, 1.28] | 1.09 [0.90, 1.31] |  | 1.29 [1.04, 1.60] | 1.04 [0.73, 1.48] |  |
| Metabolic ideal + Unfavorable lifestyle | 1.31 [1.15, 1.48] | 1.11 [0.90, 1.37] |  | 1.53 [1.21, 1.95] | 0.88 [0.58, 1.34] |  |
| Metabolic poor + Favorable lifestyle | 1.29 [1.13, 1.48] | 1.37 [1.04, 1.79] |  | 1.36 [1.05, 1.76] | 1.18 [0.70, 2.01] |  |
| Metabolic poor + Intermediate lifestyle | 1.32 [1.16, 1.49] | 1.42 [1.12, 1.80] |  | 1.43 [1.12, 1.82] | 1.17 [0.73, 1.87] |  |
| Metabolic poor + Unfavorable lifestyle | 1.55 [1.36, 1.78] | 1.52 [1.18, 1.97] |  | 1.76 [1.35, 2.28] | 1.77 [1.12, 2.79] |  |
| **Gender** | Male | Female | 0.11 | Male | Female | 0.26 |
| Metabolic ideal + Favorable lifestyle | 1.00 [Reference] | 1.00 [Reference] |  | 1.00 [Reference] | 1.00 [Reference] |  |
| Metabolic ideal + Intermediate lifestyle | 1.27 [1.10, 1.45] | 1.02 [0.89, 1.17] |  | 1.52 [1.17, 1.98] | 1.00 [0.77, 1.29] |  |
| Metabolic ideal + Unfavorable lifestyle | 1.40 [1.21, 1.62] | 1.10 [0.93, 1.31] |  | 1.65 [1.24, 2.18] | 1.04 [0.74, 1.46] |  |
| Metabolic poor + Favorable lifestyle | 1.38 [1.17, 1.64] | 1.25 [1.05, 1.48] |  | 1.51 [1.09, 2.10] | 1.16 [0.83, 1.62] |  |
| Metabolic poor + Intermediate lifestyle | 1.48 [1.28, 1.72] | 1.19 [1.00, 1.42] |  | 1.54 [1.15, 2.07] | 1.26 [0.91, 1.74] |  |
| Metabolic poor + Unfavorable lifestyle | 1.70 [1.45, 1.98] | 1.44 [1.17, 1.78] |  | 2.02 [1.50, 2.72] | 1.60 [1.09, 2.36] |  |
| **History of bowel screening** | Yes | No | 0.11 | Yes | No | 0.37 |
| Metabolic ideal + Favorable lifestyle | 1.00 [Reference] | 1.00 [Reference] |  | 1.00 [Reference] | 1.00 [Reference] |  |
| Metabolic ideal + Intermediate lifestyle | 1.08 [0.93, 1.26] | 1.17 [1.03, 1.32] |  | 1.06 [0.78, 1.43] | 1.33 [1.06, 1.68] |  |
| Metabolic ideal + Unfavorable lifestyle | 1.19 [0.99, 1.43] | 1.30 [1.13, 1.48] |  | 1.23 [0.87, 1.75] | 1.41 [1.09, 1.83] |  |
| Metabolic poor + Favorable lifestyle | 1.09 [0.90, 1.32] | 1.45 [1.25, 1.68] |  | 1.01 [0.68, 1.48] | 1.52 [1.14, 2.02] |  |
| Metabolic poor + Intermediate lifestyle | 1.18 [0.98, 1.41] | 1.44 [1.25, 1.65] |  | 1.18 [0.83, 1.68] | 1.47 [1.12, 1.93] |  |
| Metabolic poor + Unfavorable lifestyle | 1.31 [1.07, 1.61] | 1.71 [1.47, 1.99] |  | 1.41 [0.96, 2.07] | 1.98 [1.50, 2.61] |  |
| **Family history of cancer** | Yes | No | 0.88 | Yes | No | 0.55 |
| Metabolic ideal + Favorable lifestyle | 1.00 [Reference] | 1.00 [Reference] |  | 1.00 [Reference] | 1.00 [Reference] |  |
| Metabolic ideal + Intermediate lifestyle | 1.10 [0.95, 1.27] | 1.15 [1.02, 1.31] |  | 1.33 [1.00, 1.77] | 1.16 [0.91, 1.48] |  |
| Metabolic ideal + Unfavorable lifestyle | 1.11 [0.93, 1.32] | 1.35 [1.17, 1.56] |  | 1.33 [0.95, 1.85] | 1.33 [1.01, 1.74] |  |
| Metabolic poor + Favorable lifestyle | 1.39 [1.15, 1.66] | 1.26 [1.08, 1.48] |  | 1.43 [1.00, 2.03] | 1.28 [0.94, 1.74] |  |
| Metabolic poor + Intermediate lifestyle | 1.36 [1.15, 1.62] | 1.30 [1.12, 1.50] |  | 1.24 [0.88, 1.75] | 1.46 [1.11, 1.93] |  |
| Metabolic poor + Unfavorable lifestyle | 1.49 [1.24, 1.80] | 1.59 [1.35, 1.86] |  | 1.67 [1.17, 2.39] | 1.77 [1.31, 2.38] |  |

Table S3 Sensitivity analysis of the follow-up time

| Sensitivity analysis | CRC incidence | | | CRC mortality | | |
| --- | --- | --- | --- | --- | --- | --- |
|  | Hazard ratio (95% CI) | | *P-interaction* | Hazard ratio (95% CI) | | *P-interaction* |
|  | Group 1^*^ | Group 2^#^ | 0.95 | Group 1 | Group 2 | 0.87 |
| Metabolic ideal + Favorable lifestyle | 1.00 [Reference] | 1.00 [Reference] |  | 1.00 [Reference] | 1.00 [Reference] |  |
| Metabolic ideal + Intermediate lifestyle | 1.13 [1.03, 1.24] | 1.11 [1.00, 1.23] |  | 1.22 [1.02, 1.47] | 1.23 [1.01, 1.50] |  |
| Metabolic ideal + Unfavorable lifestyle | 1.25 [1.12, 1.40] | 1.24 [1.10, 1.39] |  | 1.33 [1.08, 1.64] | 1.29 [1.03, 1.62] |  |
| Metabolic poor + Favorable lifestyle | 1.30 [1.15, 1.46] | 1.27 [1.12, 1.45] |  | 1.30 [1.04, 1.64] | 1.23 [0.96, 1.59] |  |
| Metabolic poor + Intermediate lifestyle | 1.33 [1.19, 1.49] | 1.31 [1.16, 1.48] |  | 1.36 [1.09, 1.68] | 1.30 [1.03, 1.65] |  |
| Metabolic poor + Unfavorable lifestyle | 1.56 [1.38, 1.76] | 1.56 [1.37, 1.77] |  | 1.75 [1.40, 2.20] | 1.75 [1.37, 2.24] |  |

^*^Group1: the original group

^#^Group 2: excluding the first 2 years of follow-up
