# Supplementary material for: Association of modifiable lifestyle with colorectal cancer incidence and mortality according to metabolic status: prospective cohort study
Source: Front Oncol. 2023 May 30;13:1162221. doi: 10.3389/fonc.2023.1162221 (PMC10262687; doi:10.3389/fonc.2023.1162221)
Supplement: Supplementary file 5 [file Table_2.docx]

Supplement 1 Assignment Points of each items in lifestyle

|  | Operationalization of Recommendations | Points |
| --- | --- | --- |
| Physical training | **Total moderate-vigorous physical activity (MET minutes/week):** |  |
|  | n ≥ 3000 | 1 |
|  | 600 ＜ n ＜ 3000 | 0.5 |
|  | 600 ≥ n | 0 |
| Limit alcohol consumption | **Total ethanol (g/day):** |  |
|  | 0 | 1 |
|  | 0 ＜ n ≤ 28 (2 drinks) males and n ≤ 14 (1 drink) females | 0.5 |
|  | n > 28 (2 drinks) males and n > 14 (1 drink) females | 0 |
| Smoking | **Past history of smoking** |  |
|  | never smoking | 1 |
|  | previous smoking | 0.5 |
|  | current smoking | 0 |
| Diet | **Fruits and Vegetables (servings/day)** |  |
|  | n ≥ 5 | 0.5 |
|  | 3 ＜ n ＜ 5 | 0.25 |
|  | n ≤ 3 | 0 |
|  | **Grains (servings/day)** |  |
|  | n ≥ 5.5 | 0.5 |
|  | 2 ＜ n ＜ 5.5 | 0.25 |
|  | n ≤ 2 | 0 |
|  | **Red meat (servings/week)** |  |
|  | 1.5 ≥ n | 0.5 |
|  | 1.5 ＜ n ＜ 4 | 0.25 |
|  | 4 ≤ n | 0 |
|  | **Processed meat (servings/week)** |  |
|  | 1.5 ≥ n | 0.5 |
|  | 1.5 ＜ n ＜ 4 | 0.25 |
|  | 4 ≤ n | 0 |
